# Study of uterine kinetics in nonpregnant women using cine‐mode magnetic resonance imaging

**DOI:** 10.1002/rmb2.12295

**Published:** 2019-08-08

**Authors:** Akira Nakashima, Isamu Komesu, Tetsuro Sakumoto, Hakuraku Hamakawa, Yoko Terada, Hisako Takayama, Sigeru Kamiyama, Masahiro Higashi, Keiko Ishigaki, Aritoshi Nakaza, Kimio Ushijima, Yoshimitsu Tokunaga

**Affiliations:** ^1^ Soranomori Clinic Yaese Japan; ^2^ Department of Obstetrics and Gynecology Kurume University of Medicine Fukuoka Japan

**Keywords:** cine‐mode magnetic resonance imaging, embryo transfer, smooth muscle cells, uterine contraction, uterine muscle

## Abstract

**Purpose:**

To evaluate the uterine kinetics in each phase of the menstrual cycle when observed in detail using cine‐mode magnetic resonance imaging (MRI) of sagittal and transverse plane images.

**Methods:**

Seven volunteers with a history of multiple natural pregnancies and deliveries were enrolled from January 2017 to May 2017. The kinetic parameters (depth, frequency, and direction) of uterine muscle contractions were evaluated in cine‐mode MRI.

**Results:**

Strong contractions from the uterine cornua to cervix were detected during menstruation. In the late follicular phase, the frequency of opposing contractions from the cervix and uterine cornua increased. Immediately before ovulation, contractions from the cervix reached the uterine fundus. After ovulation, opposing contractions returned. These contractions gradually decreased in the mid‐luteal phase, while fine contractions from the cervix to the middle of the uterine body were frequently observed until 7 days after ovulation. Few contractions were observed in the implantation phase.

**Conclusions:**

Our data suggest that the uterine kinetics change in each phase of the menstrual cycle in accordance with the purpose of the uterus in each phase. Further, cine‐mode MRI studies of each phase are needed to assess the relationships between uterine kinetics and infertility.

## 1 INTRODUCTION

The uterus is an organ dedicated to reproduction and can vary its function within a short period of time, based on reproductive requirements. In women of reproductive age, the uterus plays a role in the induction of menstruation each month. Subsequently, the uterus regenerates the endometrium, secretes cervical mucus, and prepares the uterine environment for implantation. The pregnant uterus contributes to fetal growth and development; it also induces labor and contributes to delivery at the appropriate time.

Intrauterine pressure measurement using a transducer and transvaginal ultrasound examination has been utilized to study the kinetics of the uterus.^1^, ^2^, ^3^ Intrauterine pressure is reportedly elevated during menstruation, and the number of uterine contractions increases during the follicular phase. Ultrasound studies have revealed that endometrial wave‐like activity originates from the uterine cervix and travels toward the fundus in the follicular phase.^3^, ^4^, ^5^, ^6^

Cine‐mode magnetic resonance imaging (MRI) was recently developed and has been used for functional analysis of various organs in many clinical fields.^7^, ^8^, ^9^ In cine‐mode MRI, serial images are taken in the same plane at high speed with short intervals; these are used to produce a moving image resembling a time‐lapse video recording.

In the field of gynecology, cine‐mode MRI has reportedly been useful for kinetic analysis of uterine function during the past decade.^10^, ^11^, ^12^, ^13^, ^14^, ^15^, ^16^ Fujiwara et al^11^ reported a change in the frequency and direction of uterine peristalsis during the menstrual cycle as shown by cine‐mode MRI. Additionally, they showed a direct correlation between dysmenorrhea and uterine contraction.^11^ Yoshino et al^15^ reported that frequent endometrial movement in the implantation phase reduced the pregnancy rate in women with uterine fibroids. In addition, Orisaka et al described the normal uterine kinetics of three nonpregnant women during the menstrual cycle, as well as in women with uterine fibroids.^13^ Furthermore, Shitano et al analyzed uterine kinetics of nonpregnant women using coronal plane images; they found contractions originated from the uterine endometrium and traveled to the outer layer of the myometrium during the follicular phase.^17^

The present study aimed to further characterize uterine kinetics during each phase of the menstrual cycle in nonpregnant women with normal menstrual cycles and a history of multiple pregnancies and deliveries. Notably, our study used cine‐mode MRI performed in the sagittal and transverse planes, which has not been previously published. Furthermore, MRI in our study comprised 90 images over 3 minutes, with intervals of 2 seconds between images (ie, 30 frames per second, similar to the characteristics of television); these images were assembled into time‐lapse videos to investigate the kinetics of uterine myometrial contractions with higher frame rate than in previous reports.

## 2 MATERIALS AND METHODS

### 2.1 Volunteers

We performed the present study in the Soranomori Clinic during the period from January 2017 to May 2017. Informed consent was obtained from all volunteers, and this study was approved by our institutional review board. Seven healthy female volunteers, 30‐43 years of age, participated in this study. They had regular menstrual cycles of 26‐30 days and a history of ≥2 pregnancies and deliveries after natural conception. None of the seven women had uterine myomas, adenomyosis, or a history of recurrent miscarriages.

### 2.2 Study design

Cine‐mode MRI was performed at 2‐4 days after the beginning of menstruation, in the late follicular phase at the beginning of the luteinizing hormone (LH) surge, in the periovulatory period (2‐3 days after LH surge), and in the implantation phase (6‐10 days after ovulation). LH surge was assessed daily by using a urinary LH kit (Clearview^®︎^; Alere Medical) from the 8 day of menstruation. The serum concentrations of follicle‐stimulating hormone, LH, estradiol, and progesterone were checked (Access; Beckman Coulter, Inc) at each cine‐mode MRI examination. Transvaginal ultrasound examinations were not performed to avoid stimulating the uterus. The growth of dominant follicles was confirmed by T2‐weighted MRI.

### 2.3 Cine‐mode MRI

**Figure 1:**
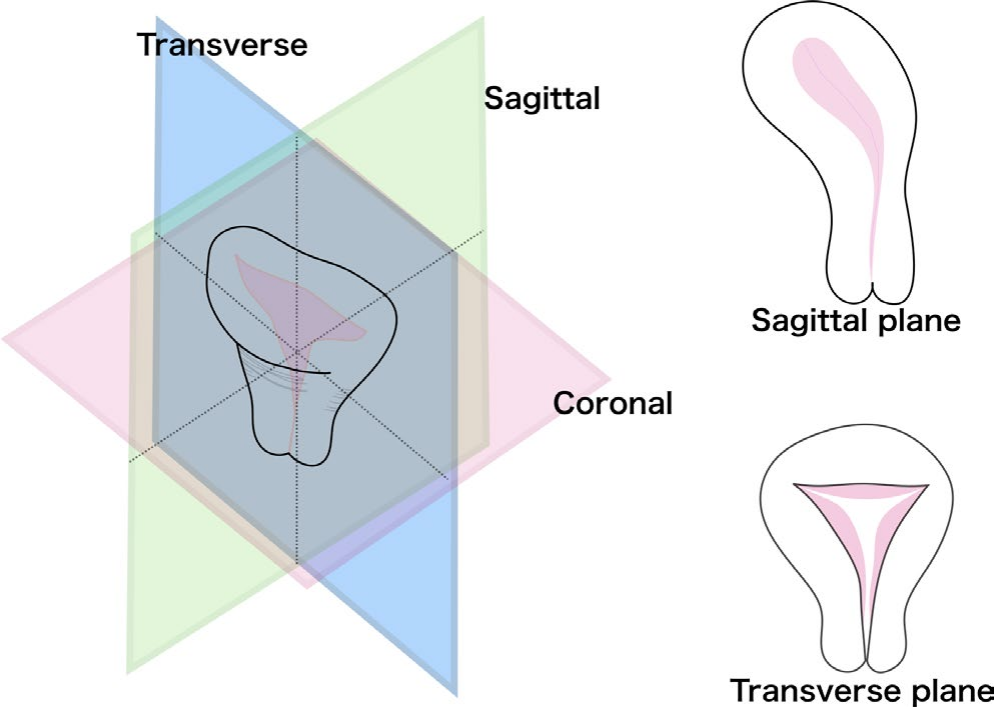
Definition of sagittal and transverse magnetic resonance imaging planes. The sagittal plane contains the mid‐fundus of the uterus and uterine cervix. The transverse plane contains the bilateral uterine cornua and cervix in a single image showing the triangular shape of the uterine cavity

MRI examinations were performed using a 1.5‐T magnet unit with a four‐channel body coil at our institution (Achieva 1.5T Conversion; Philips Japan). At the beginning of the MRI examination, pelvic images were acquired by three‐dimensional plane imaging (sagittal, coronal, and transverse plane images). The sagittal and transverse planes were then adjusted as follows, in accordance with uterine shape: the sagittal plane contained the mid‐fundus of the uterus and the uterine cervix, whereas the transverse plane contained the bilateral uterine cornua and cervix in a single image showing the triangular shape of the uterine cavity (Figure 1).

Under quiet respiration, 90 two‐dimensional images were acquired using a single‐shot turbo spin‐echo sequence (repetition time/echo time, 2000/90 ms; field of view, 250 mm; slice thickness, 6 mm; matrix, 256 × 256) every 2 seconds for 3 minutes, and the cine mode was constructed with 30 images per second. In these MRI examinations, gastrointestinal peristalsis was not suppressed with hyoscine butylbromide, as this agent can suppress uterine contractions.

### 2.4 Imaging and statistical analyses

The presence of uterine myometrial contractions, sources and terminals of uterine wave‐like contractions, frequency of peristalsis, contracting myometrial layer, and deformity of the uterine cavity were analyzed by three gynecologists and one radiological technologist, and described by their consensus. Differences in number and direction (fundus–cervix) of uterine peristaltic contractions were compared within menstrual phases by the Mann‐Whitney *U* test and between menstrual phases with the Kruskal‐Wallis test. Differences in contractile frequency (right–left) within menstrual phases were compared with the Mann‐Whitney *U* test. All statistical comparisons were performed with Ekuseru‐Toukei (Ekuseru‐Toukei 2012; Social Survey Research Information Co., Ltd.).

## 3 RESULTS

The seven volunteers had a mean age of 40.0 ± 2.0 years. Their mean cycle length was 28.0 ± 1.1 days; mean gravida was 3.29 ± 0.88, and mean parity was 3.29 ± 0.88. Notably, all volunteers had a history of two or more natural pregnancies without undergoing infertility treatment. The serum concentrations of each hormone at each of the five examinations are summarized in Table 1.

**Table 1:** Volunteers’ serum hormone concentrations

	Menstruation (n = 7), mean ± SD	Late follicular phase (n = 4), mean ± SD	LH surge (n = 2), mean ± SD	Postovulatory period (n = 3), mean ± SD	Implantation phase (n = 4), mean ± SD
Follicle‐stimulating hormone (mIU/mL)	10.05 ± 4.48	––a	––	––	––
Luteinizing hormone (mIU/mL)	5.07 ± 2.81	10.80 ± 3.21	40.54 ± 10.97	––	––
Estradiol (pg/mL)	33.57 ± 14.28	297.00 ± 95.77	356.0 ± 24.0	62.33 ± 15.46	181.00 ± 103.04
Progesterone (ng/mL)	0.40 ± 0.18	0.45 ± 0.15	0.86 ± 0.13	3.74 ± 0.97	11.30 ± 4.46

Abbreviations: LH, luteinizing hormone; SD, standard deviation.

a Dash indicates measurement was not performed.

**Figure 2:**
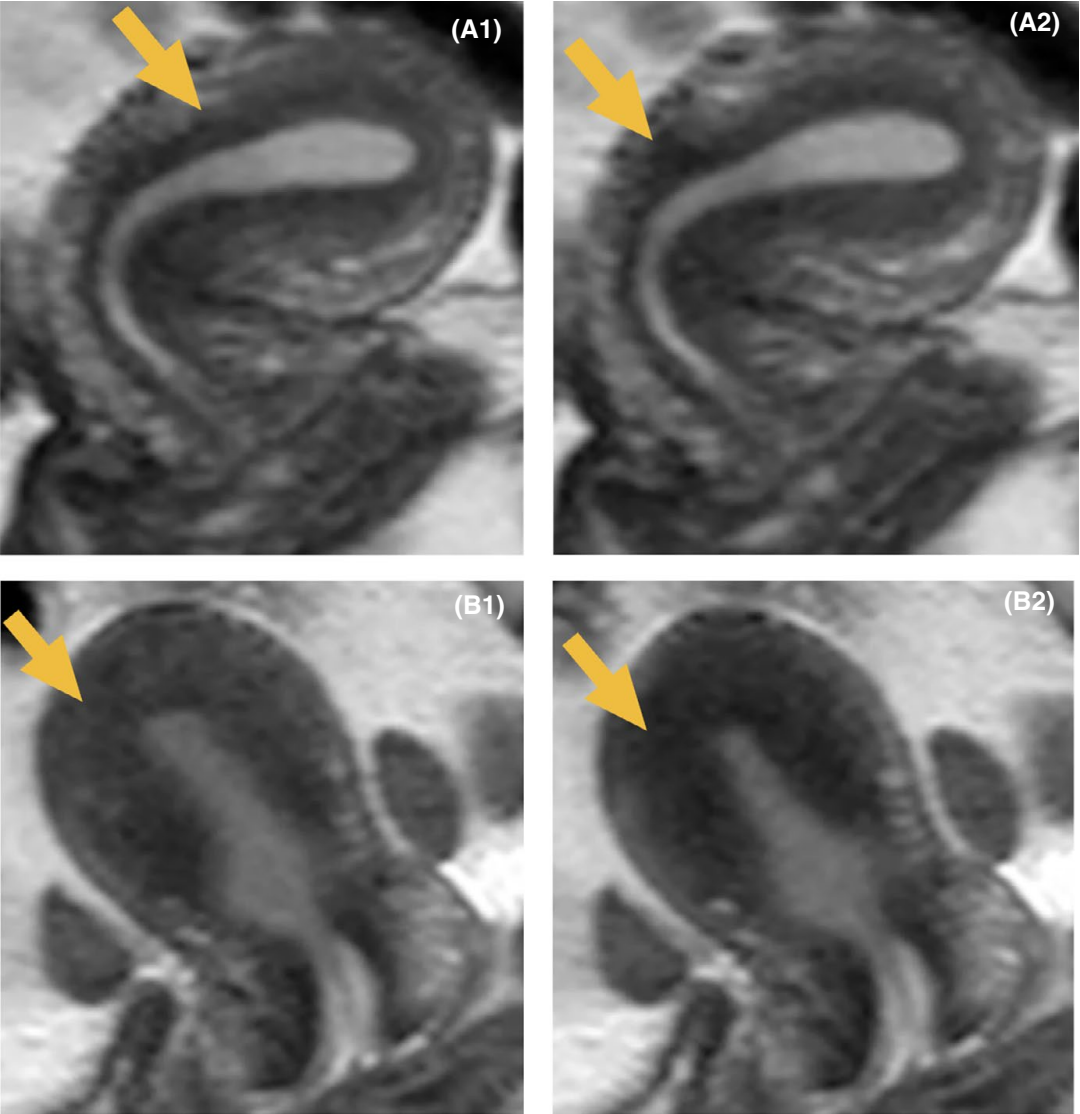
Magnetic resonance image of uterine contraction. The arrow shows the contraction area. The contraction site is detected as a low‐intensity area. Shifting of the arrow (from a‐1 to a‐2) represents continuous movement of the contraction area in the uterine myometrial layer. In b‐1 and b‐2, three‐fourths of the inner side of the uterine myometrium contracted

**Figure 3:**
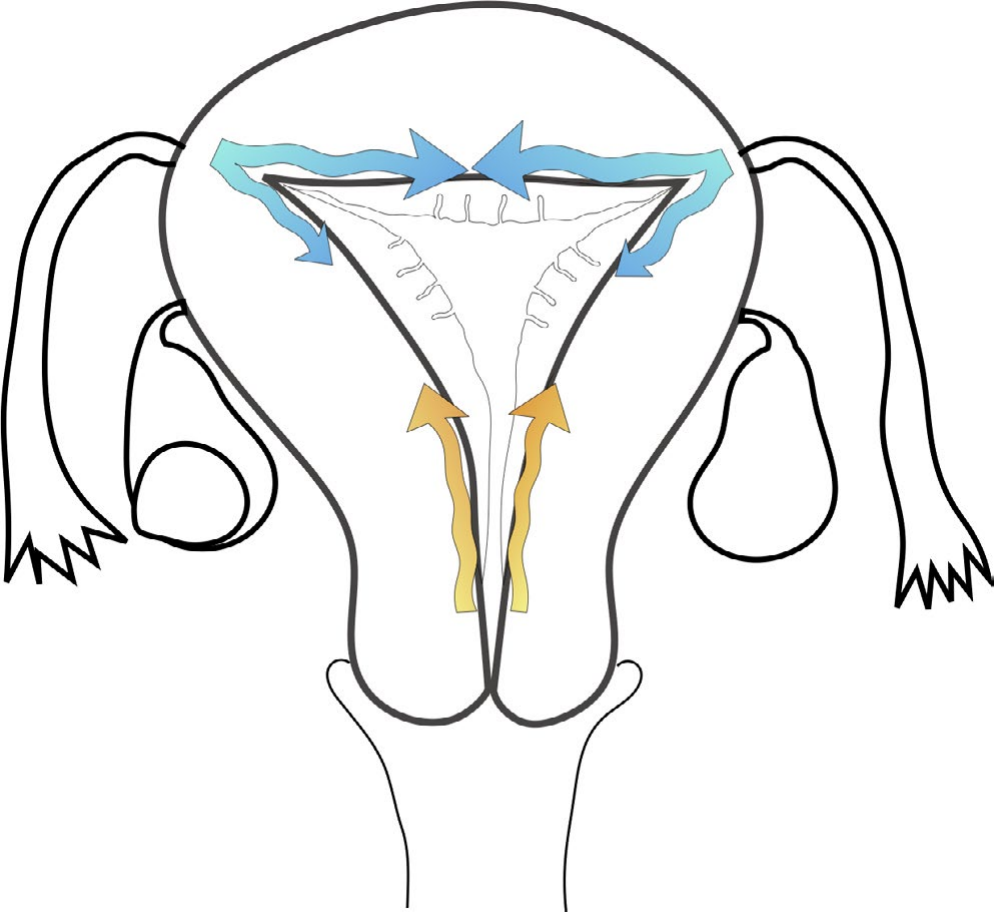
Schematic presentation of wave‐like contraction. The wave‐like contraction that arose at the cervix reached the uterine fundus, whereas waves that arose at the bilateral uterine cornua reached the cervix and opposite cornu (arrows)

Contraction of the uterine myometrial layer was detected as a low‐intensity area in T2‐weighted images (Figure 2). Continuous acquisition of the images at high speed and subsequent production of the moving image enabled visualization of the movement as a wave‐like contraction (Figure 3). Two directions of wave‐like contraction were detected based on specific menstrual phases. One arose at the cervix and reached the uterine fundus, while the other arose at the bilateral uterine cornua and reached the cervix and the opposite cornu. The directions and frequencies of these movements varied depending on the menstrual phase. In addition, the depth of the contracting myometrial layer changed in each phase. Two types of contractions were observed: contraction of the inner‐side myometrium immediately below the junctional zone (Figure 1a1,a‐2) and a wide layer of contraction around three‐fourths of the inner‐side myometrium (Figure 1b1,b‐2). Table 2 shows the mean frequencies of uterine contractions from the uterine fundus to the cervix and from the cervix to uterine fundus, as well as the depth of the contracting uterine myometrium layer in the sagittal plane. Table 3 shows the mean frequencies of uterine contractions from the bilateral cornua to the cervix and from the cervix to the bilateral cornua, as well as the depth of the contracting uterine myometrium layer in the transverse plane. All of the contractions from the cervix to the bilateral cornua were synchronized. On the other hand, the contractions from the bilateral cornua didn't synchronize in the transverse plane. In the case showed a difference in the frequency of the contraction between the bilateral cornua, the larger number was defined as the number of the contractions from the fundus (Table 3).

**Table 2:** Characteristics of wave‐like contractions in each phase in the sagittal plane

	F → C, mean ± SD^a^	C → F, mean ± SD	*P* (direction)^b^	Thickness^d^
Menstruation (n = 7)	1.29 ± 0.88	0.86 ± 0.64	.3927	3/4,1/4,3/4,2/4,3/4,4/4,4/4
Late follicular phase (n = 4)	5.00 ± 1.22	4.00 ± 2.74	.6612	JCZ (all)
LH^e^ surge (n = 2)	0.0	6.50 ± 0.50	.1025	JCZ (all)
Postovulatory period (n = 3)	4.33 ± 2.62	6.00 ± 2.16	.5002	JCZ (all)
Implantation phase (n = 3)	0.25 ± 0.43	0.00	.3173	JCZ/Isthmus6, Isthmus4^e^
*P* (phase)^c^	.0044	.0051		

Abbreviations: LH, luteinizing hormone; JCZ, junctional zone; SD, standard deviation.

a F → C, number of wave‐like contractions from uterine fundus (cornu) to cervix; C → F, number of wave‐like contractions from uterine cervix to fundus.

b Mann‐Whitney *U* test.

c Kruskal‐Wallis test.

d Thickness, ratio of myometrial layer showing contraction; Isthmus, fine contractions around cervical isthmus (number indicates contractions per 3 min).

e Only two thickness measurements were performed among the three volunteers.

**Table 3:** Characteristics of wave‐like contractions in each phase in the transverse plane

	F → C, mean ± SD^a^	C → F, mean ± SD	*P* (direction)^b^	Thickness^d^
Menstruation (n = 7)	1.29 ± 0.45	1.00 ± 1.41	.2235	2/4,1/4,4/4,2/4^e^
Late follicular phase (n = 4)	5.75 ± 1.79	2.75 ± 1.92	.1391	JCZ (all)
LH Surge (n = 2)	0.50 ± 0.50	7.50 ± 0.50	.1213	JCZ (all)
Postovulatory period (n = 3)	4.00 ± 2.83	4.00 ± 2.16	.8166	JCZ (all)
Implantation phase (n = 3)	1.00 ± 0.71	0.25 ± 0.43	.1547	JCZ, JCZ/Isthmus7^d^, JCZ/Isthmus5
*P* (phase)^c^	.0087	.0258		

Abbreviations: LH, luteinizing hormone; JCZ, junctional zone; SD, standard deviation.

a F → C, number of wave‐like contractions from uterine fundus (cornu) to cervix; C → F, number of wave‐like contractions from uterine cervix to fundus.

b Mann‐Whitney *U* test.

c Kruskal‐Wallis test.

d Thickness, ratio of myometrial layer showing contraction; Isthmus, fine contractions around cervical isthmus (number indicates contractions per 3 min).

e Only four thickness measurements were performed among the seven volunteers.

### 3.1 Characteristics of contractions in each phase

#### 3.1.1 Menstruation phase

The contractions arising from the uterine cornua occurred with similar frequency to those arising from the cervix during the menstruation phase in both sagittal and transverse planes (*P* = .3927, *P* = .2235). With respect to the depth of myometrial contraction layer, one‐half to three‐fourths of the inner‐side myometrial layer contracted strongly in five of the seven volunteers; in the remaining two volunteers, the entire myometrial layer contracted strongly.

#### 3.1.2 Late follicular phase

The serum estrogen concentration rose acutely as the follicle grew in the late follicular phase (Table 1). During this phase, uterine myometrial contractions occurred only in the inner‐side myometrial layer immediately below the junctional zone. Contractions with gradually increasing frequency were then detected both from the uterine cornua and cervix. The contractions arising from the uterine cornua occurred with similar frequency to those arising from the cervix during the late follicular phase in both sagittal and transverse planes (*P* = .6612, *P* = .1391; Tables 2 and 3). Contractions arising from the uterine cornua ipsilateral to the leading follicle did not show a significant difference in frequency relative to those arising from the contralateral side (*P* = .1081; Table 4). Wave‐like contractions from both the cervix and cornua seemed to counteract each other and disappear around the middle region of the uterine body when an opposing contraction was encountered.

**Table 4:** Differences of wave‐like contractions in late follicular phase and during ovulation in the transverse plane

	R → L, mean ± SD^a^	L → R, mean ± SD^a^	Derivation of contraction		*P* (derivation)^c^
			Ipsilateral, mean ± SD^b^	Contralateral, mean ± SD^b^	
Late follicular phase (n = 4)	3.75 ± 2.49	4.50 ± 3.04	5.75 ± 1.79	2.50 ± 2.69	.1081
Postovulatory period (n = 3)	2.67 ± 0.94	2.67 ± 3.77	4.00 ± 2.83	1.33 ± 1.89	.2612

Abbreviation: SD, standard deviation

a R → L, the number of wave‐like contraction from the right cornu to left cornu; L → R, the number of wave‐like contraction from left cornu to the right cornu.

b Ipsilateral, frequency of wave‐like contractions from the uterine cornu of the dominant follicle; Contralateral, frequency of wave‐like contractions from the uterine cornu of the nondominant follicle.

c Mann‐Whitney *U* test.

#### 3.1.3 LH surge phase

In the LH surge phase, the frequency of wave‐like contractions from the cervix peaked and reached the uterine fundus in the both of the sagittal plane and the transverse plane (*P* = .1025, *P* = .1213; Tables 2 and 3). In contrast, the contractions from the bilateral uterine cornua were nearly undetectable.

#### 3.1.4 Postovulatory period

After ovulation, the LH surge ended, the serum estradiol concentration decreased, and the progesterone concentration began to rise. In this phase, wave‐like contractions from the uterine cornua appeared again. The frequency of contractions from the uterine cervix decreased after the LH surge. The contractions arising from the uterine cornua occurred with similar frequency to those arising from the cervix during the late follicular phase in both sagittal and transverse planes (*P* = .5002, *P* = .8166; Tables 2 and 3). Contractions arising from the uterine cornua ipsilateral to the ovulated ovary did not show a significant difference in frequency relative to those arising from the contralateral side (*P* = .2612; Table 4).

#### 3.1.5 Implantation phase

A gradually increasing frequency of wave‐like contractions from the uterine cervix to the middle of the uterine body was observed around the implantation period. These contractions appeared as fine movements immediately below the junctional zone, and occasionally reached the uterine fundus. Few contractions from the uterine cornua were observed. At approximately 8‐9 days after ovulation, no contractions were detected from the cervix or uterine cornua. As the progesterone concentration began to decrease, slight contractions from the uterine cornua began to appear in one volunteer.

#### 3.1.6 Overall frequencies of contraction among the five phases

The frequencies of contractions from the uterine fundus to the cervix and from the cervix to the uterine fundus significantly differed among the five phases in the sagittal plane (*P* = .0044, *P* = .0051) and in the transverse plane (*P* = .0087, *P* = .0258).

## 4 DISCUSSION

The derivation, strength, frequency, and myometrial layer of uterine contractions might change for various reasons during the menstrual cycle. The present study revealed potential variations in contractions during the menstrual cycle, although the differences were not statistically significant. Uterine movements can be vaguely detected using transvaginal ultrasound tomography, whereas cine‐mode MRI enables detailed visualization of uterine contractions.^10^, ^11^, ^14^ Cine‐mode MRI may thus be more beneficial than ultrasound imaging for precise detection and confirmation of the direction of uterine contractions.^18^

Cine‐mode MRI is preferable to elucidate the derivation, frequency, and direction of uterine myometrial movement. In a cine‐mode MRI study using the coronal plane, Shitano et al 17 captured a total of 60 images at 3‐second intervals and used these to assemble a time‐lapse video of 18 seconds in length. Orisaka et al similarly captured a total of 32 images at 6‐second intervals and used these to assemble an 8‐second time‐lapse video.^13^ These studies showed the frequency, direction, and outer myometrium contractions during uterine peristalsis. Importantly, our study used cine‐mode time‐lapse videos comprising 90 images captured at 3‐second intervals. Therefore, the present study showed uterine movements in greater detail in both sagittal and transverse planes. In particular, transverse plane examination revealed uterine peristalsis from bilateral cornua and the cervix by adjusting the plane images to the uterine coronal axis.

Our findings are consistent with those of previous studies in which transvaginal ultrasound and cine‐mode MRI indicated the direction of uterine contractions during menstruation.^13^, ^16^, ^18^ In the present study, observation of uterine contraction during the follicular phase was performed at two different time points: prior to the LH surge phase (late follicular phase) and during the LH surge phase. Our findings revealed two different contractions from the uterine cornua to the cervix and from the cervix to the fundus during the late follicular phase, before LH surge. Orisaka et al and Shitano et al described these opposing movements before LH surge using sagittal and coronal images of the late follicular phase.^13^, ^17^ Shitano et al reported that peristalsis from the contralateral side toward the cornu of the ovulation side was observed in only three of 10 cases; the remaining seven showed peristalsis from the ovulation side toward the cornu of the contralateral side. Our study results were consistent with these findings, although they were not statistically significant. It has been presumed that the peristalsis toward to the ovulation side cornu was higher than toward to the contralateral side, based on a study of Hysterosalpingoscintigraphy with microspheres 5; therefore, further investigations are needed.

At the time of the LH surge, the frequency of the uterine wave‐like movements from the cervix reaches its peak, and the wave reaches the uterine fundus. The movements from the uterine cornua become undetectable; frequent wave‐like movements from the cervix might counteract them.

Shortly after the LH surge ended in this study, the frequency of movements from the cervix decreased and movements from the uterine cornua became detectable again. In the transverse plane, the frequency of the contraction from the ovulation side cornu tended to be higher than that from the contralateral side, as observed before ovulation; however, this was not statistically significant.

The frequency of wave‐like movements from the uterine cervix to the uterine fundus gradually decreased after ovulation. However, fine movements from the cervix frequently occurred around the cervical isthmus only during this period. Orisaka et al reported peristaltic movement of the cervix isthmus during the implantation phase in nonpregnant women.^13^ Our study found this peristalsis of the cervical isthmus, which disappeared in two of four volunteers in the middle of the luteal phase. In the same period, the frequency of the wave‐like movements from the uterine cornua also decreased. The decreased frequency of movements from the uterine cornua is consistent with previous studies involving intrauterine pressure measurements, transvaginal ultrasound, and cine‐mode MRI.^2^, ^6^, ^13^, ^15^, ^16^

Based on reduction in sex steroid hormone concentrations, the uterine myometrium in nonpregnant women gradually begins to contract from the uterine cervix or cornua 10 days after ovulation. Prior to the beginning of menstruation, all layers of the uterine myometrium begin to sporadically contract.

These phenomena suggest that sex steroid hormones may have important roles in nonpregnant uterine contractions. Estrogen reportedly promotes the formation of gap junctions between uterine smooth muscle cells to enhance the transmission of muscle contractions, while progesterone suppresses the formation of gap junctions and plays a role in quiescing the uterine muscle.^19^, ^20^, ^21^ Estrogen might lead to contractions of the uterine muscle layer along with follicle development, and the frequency of uterine contractions gradually decreases after ovulation. When the serum concentration of progesterone decreases, the smooth muscle tissues begin to gradually react to the electric stimulus again, and menstruation might begin. Notably, the administration of an anti‐progesterone drug (RU486) has been reported to promote the reaction of uterine muscle to prostaglandin, thereby inducing uterine contraction.^22^ Furthermore, recent reports indicate that telocytes were found in the uterine myometrium and endometrium; these cells might regulate uterine contractions in various stages of the menstruation cycle, pregnancy, labor, and postpartum uterine involution.^23^, ^24^ Sex steroid hormones are presumed to influence smooth muscle cells and telocytes, and the kinetics of the uterus may change during the menstrual cycle. Further physiological research is required in this field.

It was difficult to fully clarify the trends of uterine kinetics in this study because of the small number of volunteers. Although the ethics committee approved a larger study, we were unable to recruit additional volunteers. In addition, the examinations were not performed every point during the menstrual cycle in all volunteers. Because this was a small study, we did not exclude those participants from the analysis because of missing data. However, this study has shown several new aspects of the uterine kinetics in the menstrual cycle of nonpregnant women. Further studies should involve a larger sample size and examinations at multiple points in the menstrual cycle.

The frequency, intensity, and direction of uterine myometrial contractions change throughout the menstrual cycle. Variations in these movements are essential for reproduction. It is necessary to investigate the mechanisms of these variations in detail and to apply cine‐mode MRI to elucidate the causes of gynecologic disease, infertility, and obstetric complications.

## DISCLOSURES

*Conflict of interest*: The authors declare no conflict of interest. Human rights statements and informed consent: All the procedures were followed in accordance with the ethical standards of the responsible committees on human experimentation (institutional and national) and with the principles of the Helsinki Declaration of 1964 and its later amendments. The protocol of this research project was approved by the institutional review board of Soranomori Clinic. Informed consent was obtained from all volunteers for the examinations and in this study. Additional informed consent was obtained from all volunteers for which identifying information is included in this article. Animal studies: This article does not contain any studies with animal subjects performed by the any of the authors.
